# Capacity of a Microbial Synbiotic To Rescue the *In Vitro* Metabolic Activity of the Gut Microbiome following Perturbation with Alcohol or Antibiotics

**DOI:** 10.1128/aem.01880-22

**Published:** 2023-02-22

**Authors:** Braden T. Tierney, Pieter Van den Abbeele, Gabriel A. Al-Ghalith, Lynn Verstrepen, Jonas Ghyselinck, Marta Calatayud, Massimo Marzorati, Azza A. Gadir, Brendan Daisley, Gregor Reid, Peter A. Bron, Dirk Gevers, Raja Dhir, Sheri L. Simmons

**Affiliations:** a Seed Health, Los Angeles, California, USA; b ProDigest BV, Ghent, Belgium; c CMET, Ghent, Belgium; d The University of Western Ontario, London, Ontario, Canada; e Lawson Health Research Institute, London, Ontario, Canada; Centers for Disease Control and Prevention

**Keywords:** short-chain fatty acid, gut microbiome, microbial intervention, shallow shotgun sequencing, metabolomics, cross-feeding

## Abstract

The human gut microbiome contributes crucial bioactive metabolites that support human health and is sensitive to perturbations from the ingestion of alcohol and antibiotics. We interrogated the response and recovery of human gut microbes after acute alcohol or broad-spectrum antibiotic administration in a gut model simulating the luminal and mucosal colonic environment with an inoculated human microbiome. Both alcohol and antibiotic treatments reduced the production of major short-chain fatty acids (SCFAs) (acetate, propionate, and butyrate), which are established modulators of human health. Treatment with a microbial synbiotic restored and enhanced gut function. Butyrate and acetate production increased by up to 29.7% and 18.6%, respectively, relative to untreated, dysbiotic samples. In parallel, treatment led to increases in the relative abundances of beneficial commensal organisms not found in the synbiotic (e.g., Faecalibacterium prausnitzii and the urolithin-producing organism Gordonibacter pamelaeae) as well as species present in the synbiotic (e.g., Bifidobacterium infantis), suggesting synergistic interactions between supplemented and native microorganisms. These results lead us to conclude that functional shifts in the microbiome, evaluated by both metabolite production and specific taxonomic compositional changes, are an appropriate metric to assess microbiome “recovery” following a dysbiosis-inducing disruption. Overall, these findings support the execution of randomized clinical studies to determine whether a microbial synbiotic can help restore microbiome function after a disruption.

**IMPORTANCE** The human gut microbiome is sensitive to disruptions by common stressors such as alcohol consumption and antibiotic treatment. In this study, we used an *in vitro* system modeling the gut microbiome to investigate whether treatment with a microbial synbiotic can help restore microbiome function after stress. We find that a complex gut community treated with alcohol or antibiotics showed reduced levels of production of short-chain fatty acids, which are critical beneficial molecules produced by a healthy gut microbiota. Treatment of stressed communities with a microbial synbiotic resulted in the recovery of SCFA production as well as an increase in the abundance of beneficial commensal organisms. Our results suggest that treatment with a microbial synbiotic has the potential to restore healthy gut microbiome function after stress and merits further investigation in clinical studies.

## INTRODUCTION

Gut microbes play manifold roles in modulating human health, from aiding in immune system development to regulating the nervous system (1–3). However, they are sensitive to disruption due to environmental factors like lifestyle changes, emotional stress, dietary shifts, alcohol use, and antibiotic use (4–8). These disturbances can disrupt the relationship between gut microbes and their hosts, yielding what is commonly referred to as “dysbiosis” (9). Often, a dysbiotic gut contains a surfeit of pathogenic or otherwise detrimental bacteria and can result in a variety of pathologies like diarrhea (10, 11).

Antibiotic treatment can be associated with deleterious side effects on digestive health, many of which are linked to a dysbiotic microbiome composition (12). These include diarrhea, nausea, and vomiting (13, 14). Shifts in the composition of the fecal microbiome after antibiotic treatment can last for months, with dysbiosis characterized by the underrepresentation of critical species like short-chain fatty acid (SCFA) producers (15–17). SCFAs are produced by the breakdown of dietary polysaccharides and, among other functions, can prevent chronic inflammatory states by inhibiting the production of proinflammatory cytokines, regulating T-cell differentiation, and protecting colonic epithelial cells (18–20). For example, ceftriaxone treatment has been associated with a loss of gut barrier integrity due to SCFA depletion (17). Ciprofloxacin and metronidazole, commonly administered in combination for the treatment of gastrointestinal infections, decrease the relative abundances of members of the SCFA-producing genera *Faecalibacterium*, *Subdoligranulum*, and *Roseburia* (21).

Microbiome compositional shifts also occur with alcohol consumption and in parallel to downstream deleterious effects. Alcohol use has been linked to an increased risk of liver injury, colon cancer, diarrhea, gut inflammation, and loss of gut barrier integrity (22–24). Dysbiotic microbiome shifts and bacterial overgrowth can also occur with alcohol consumption; these include blooms of *Proteobacteria* alongside losses of the SCFA-producing organisms Faecalibacterium prausnitzii and Akkermansia muciniphila, which are associated with reducing inflammation and improving barrier integrity (25–27). Previous studies have hypothesized that these microbiome changes may drive or exacerbate the effects of alcohol usage on the host, increasing inflammation and tissue damage (25, 28). For example, the intestinal microbiota from patients with severe alcoholic hepatitis (AH) has been shown to increase liver inflammation in ethanol (EtOH)-fed mice compared to the microbiota transferred from patients without AH (29). The intestinal microbiota isolated from patients with advanced alcoholic hepatitis has reduced levels of F. prausnitzii and A. muciniphila (29, 30). Microbial fermentation of ethanol has been hypothesized to increase the risk of colon cancer via acetaldehyde production (31, 32).

While the literature demonstrates that both alcohol and antibiotic usage impact the gut microbiome, the role of these changes in the body’s response to both perturbations, as well as the recovery of a healthy gut microbiota (33), is still not well understood. In addition to SCFA production, there are multiple possible microbial mechanisms that impact how individuals respond to these interventions, including ethanol detoxification and epithelial barrier repair (34, 35). The discovery of consistent dysbioses associated with these perturbations has suggested that the introduction of an exogenous healthy gut microbiota could have health benefits.

As a result, there have been both observational and randomized studies to evaluate the efficacy of microbial supplementation in restoring microbiome composition and/or function following dysbiosis due to disruption by alcohol or antibiotics (36, 37). In a small phase 1 study, fecal microbiota transplant (FMT) treatment reduced alcohol cravings, boosted SCFA levels, and reduced inflammatory cytokines in a population of patients with alcohol-related cirrhosis (38). A randomized study involving 66 alcoholic patients taking a short-term oral treatment of Bifidobacterium bifidum (strain not reported) and Lactiplantibacillus plantarum 8PA3 reported enhanced microbiota restoration and greater symptom improvement in alcohol-induced liver injury than achieved with standard therapy alone (39). Other studies have shown that probiotic supplementation ameliorates antibiotic side effects when treating infection, reduces the burden of antibiotic-induced diarrhea, and removes antibiotic-driven differences in infant microbiome compositions (14, 40–42).

In this small, *in vitro* pilot study, we measured the response of a healthy human gut microbial community to synbiotic treatment following both antibiotic- and alcohol-induced dysbiosis. The consensus scientific definition of a synbiotic is “a mixture comprising live microorganisms and substrate(s) selectively utilized by host microorganisms that confers a health benefit on the host” (43). Our goal was to determine if synbiotic treatment could restore microbial community function (as measured by SCFA production) without the promotion of potentially deleterious bacteria. Overall, we aimed to determine if the results could justify a large-scale, placebo-controlled human trial to investigate the same question in a clinical setting. Using a batch-fermentation-based gut model, we quantified shifts in microbial taxonomic ecology and SCFA contents following gut microbiome perturbation.

## RESULTS

### Measuring the impact of alcohol or antibiotics on microbial community composition and SCFA production.

**FIG 1:**
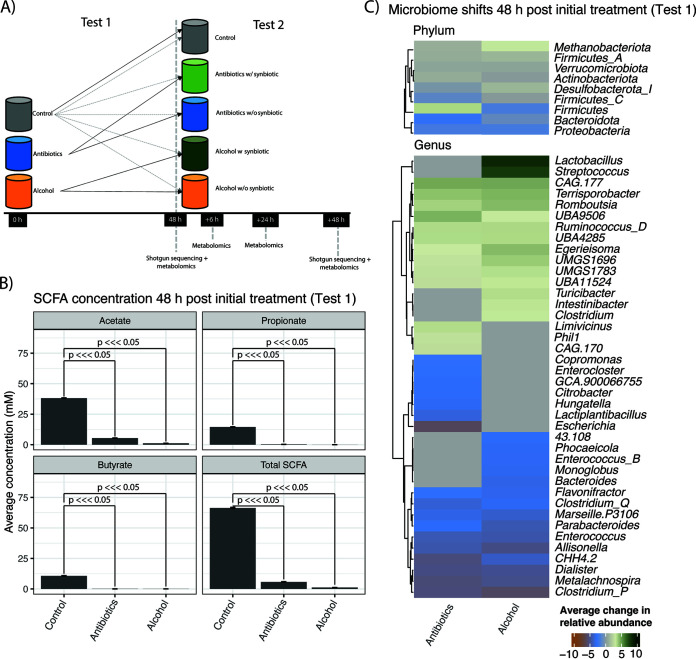
Experimental overview and microbial stress response. (A) Experimental design. Fecal matter from a healthy individual was used to inoculate triplicate fermentations in the presence of alcohol (EtOH) or antibiotics (48-h treatment) and compared to that of a control without intervention. In the second phase of the experiment, recovery after alcohol or antibiotic treatment was assessed. Colonic suspensions from both interventions were incubated for an additional 48 h in the absence of synbiotics (Antibiotics w/o synbiotic and Alcohol w/o synbiotic) or the presence of synbiotics (Antibiotics w synbiotic and Alcohol w synbiotic), while the control fermentation continued. To simulate the backup function of the mucosal environment, mucin-coated carriers from the control in phase 1 of the experiment were transferred to all colon reactors in phase 2. This is represented by the dotted gray lines. (B) Changes in SCFA concentrations as a function of antibiotic and EtOH treatment (test 1 in panel A). (C) Top statistically significant phylum- and genus-level changes (compared to the controls) in average microbial relative abundances for reactors exposed to stressors (test 1 in panel A). Gray cells in either of the columns describing relative abundance shifts indicate a non-statistically significant association (i.e., *P* value of >0.05). Green indicates enrichment relative to the control, while blue/orange indicates depletion relative to the control.

We conducted a two-phase experiment using a simulated gut ecosystem inoculated with a complex gut microbial community from a healthy donor (see Materials and Methods). In the first phase (Fig. 1A, test 1), a fecal sample was used to inoculate nine parallel reactors (three conditions with three replicates per condition). Fermentors were incubated for 48 h with either 50 μg/mL metronidazole and 30 μg/mL ciprofloxacin to mimic antibiotic treatment or 30% (vol/vol) commercial vodka to mimic alcohol consumption or were left untreated as a control. In the second phase (Fig. 1A, test 2), we assessed the recovery of the three luminal microbial communities (untreated control, antibiotic treated, and alcohol treated) by reinoculation into fresh reactors with and without the addition of a synthetic microbial consortium. In total, five conditions were tested in this second phase, each in triplicate (antibiotic with or without synbiotic, alcohol with or without synbiotic, and control). Mucin-coated beads, meant to simulate a healthy mucosal microbiota (44), were additionally transferred from the test 1 control reactors to all test 2 reactors.

Both antibiotic and alcohol treatment incubations led to substantial changes in community composition and function. The levels of all tested SCFAs dropped compared to those in control technical replicates, which remained stable throughout the 48-h incubation (Fig. 1B). We observed statistically significant (adjusted *P* value of <0.05 via a generalized linear model [see Table S1 in the supplemental material]) changes in the relative abundances of different clades (Fig. 1C). We additionally recomputed all models with alternative relative-abundance metrics (see Materials and Methods) and saw similar results (Table S2). There was an overall loss of *Proteobacteria* and *Bacteroidota*, while *Firmicutes* were enriched in the antibiotic treatment and *Methanobacteria* were enriched in the alcohol treatment. Specific genera that were decreased under both conditions included *Parabacteroides*, *Enterococcus*, *Dialister*, *Citrobacter*, and Escherichia. Antibiotics uniquely reduced the abundances of *Flavonifractor* and *Bacteroides* and increased the relative abundances of various genera, including *Lactobacillus*, *Turicibacter*, Streptococcus, and *Clostridium*, the last of which contains a variety of human pathogens (10). Alcohol supplementation also altered a variety of diverse genera, decreasing, for example, *Citrobacter* and *Lactiplantibacillus*.

### Rescue of gut metabolite levels after alcohol- and antibiotic-induced dysbiosis.

**FIG 2:**
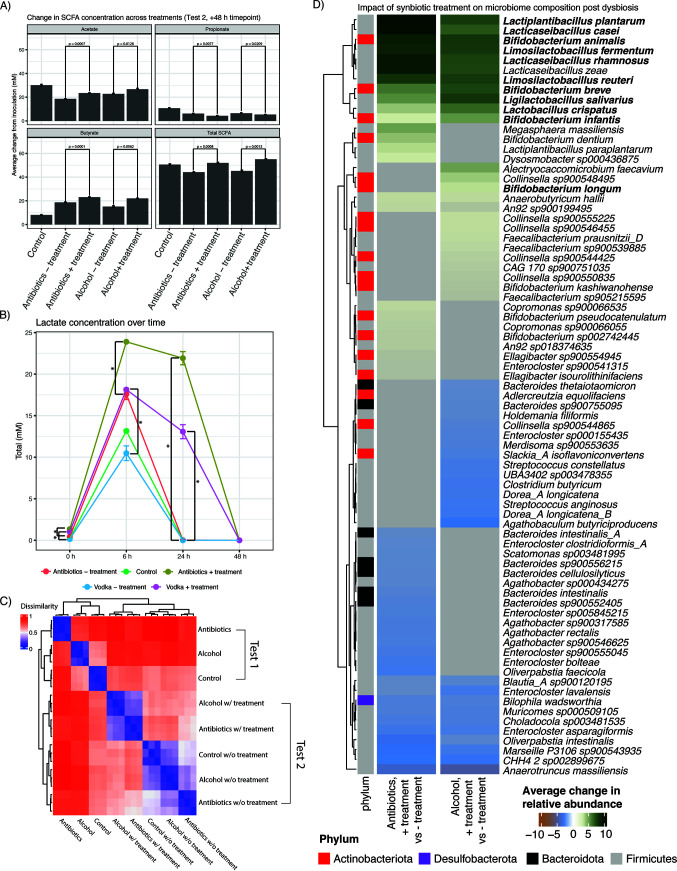
Response of dysbiotic microbiomes to treatment. (A) Changes in SCFA concentrations across treatment groups. (B) Changes in lactate concentrations over time. (C) Beta diversity values between all reactors with and without stressors and/or synbiotic treatment. (D) Top statistically significant changes (*P* value of <0.05) in average relative microbial abundances when comparing the reactor with only a stressor (after 48 h of incubation) (Fig. 1A, test 2, control) to the reactor with a stressor and synbiotic treatment (Fig. 1A, test 2). Green indicates taxa that were increased with synbiotic treatment, while blue indicates taxa that were decreased with synbiotic treatment. Gray cells in either of the columns describing abundance shifts indicate a non-statistically significant association. Species in boldface type are those present in the microbial synbiotic used.

Treatment with the synbiotic over a 48-h window increased SCFA production (*P* value of <0.05 for all comparisons described below) (Fig. 2A). By the final time point, total SCFA production in samples treated with dysbiotic stressors (Fig. 1A, test 1) was reduced by 10.7% relative to the uninoculated controls, whereas total SCFA production in samples treated with stressors and synbiotic supplementation (Fig. 1A, test 2) was increased by 5.1%. In samples exposed to a dysbiotic stressor with and without a synbiotic present (Fig. 1A, test 2), supplementation increased butyrate levels by 18.3% in the antibiotic treatment and 29.7% in the alcohol treatment. Synbiotic supplementation increased acetate levels by 18.6% in the antibiotic treatment and 13.4% in the alcohol treatment. Finally, synbiotic supplementation increased propionate levels by 30.4% in the antibiotic treatment and 16.2% in the alcohol treatment.

Lactate production after treatment with dysbiotic stressors was also altered as a function of synbiotic supplementation. After 24 h, only samples treated with the synbiotic consortium showed detectable lactate levels (13.1 to 21.9 mM) compared to the control or non-microbial-treated samples (Fig. 2B). By the end of the incubation, lactate levels had decreased in all samples.

### Alterations in microbial community composition following dysbiosis and microbial synbiotic treatment.

We next compared the impacts of synbiotic treatment on dysbiotic microbial communities. By comparing the overall beta diversity values (Fig. 2C) between experimental treatments, we observed that reactors after treatment with stressors and/or a synbiotic had three discrete microbiome signatures. The first cluster contained samples treated only with stressors and incubated for 48 h (Fig. 1A, test 1, and Fig. 2C, top 3 rows). Samples from test 2 formed the other two clusters, which were associated with the presence or absence of synbiotic supplementation. One of these contained samples treated with stressors and supplemented with the synbiotic, sampled after 48 h of incubation (Fig. 2C, middle 2 rows). The other test 2 cluster contained samples treated with stressors and not supplemented with a synbiotic, sampled after 48 h (Fig. 2C, bottom 3 rows). Note that the microbiome composition of the test 2 control reactor diverged from the microbiome composition of the test 1 untreated control due to the presence of precolonized mucin-coated beads in the test 2 control reactor.

This beta diversity analysis did not demonstrate “recovery” as defined by a return to the initial community composition prior to stress induction for either stressor. Instead, both antibiotic- and alcohol-treated communities treated with synbiotic showed similar endpoint signatures distinct from their dysbiotic states in test 1 (Fig. 2C). The observed convergence in microbiome signatures as a function of treatment with a stressor was driven predominantly by increases in SCFA- and lactic acid-producing species, some of which were members of the synbiotic consortium (Fig. 2D). These included a variety of strains of *Bifidobacterium*, a genus that contains many acetate producers, and species within the genus *Lacticaseibacillus*. Out of the 12 species (comprising 24 isolates) present in the microbial consortium, the relative abundances of 11 (species in boldface type in Fig. 2D) were still increased after 48 h in the samples previously exposed to either antibiotics or alcohol. Notably, of these, the abundances of acetate-producing species (Bifidobacterium animalis and Bifidobacterium breve) and lactic acid-producing species (Lacticaseibacillus rhamnosus, Lacticaseibacillus casei, Lactobacillus crispatus, Limosilactobacillus fermentum, Limosilactobacillus reuteri, Ligilactobacillus salivarius, and Lactiplantibacillus plantarum) in the probiotic consortium were all significantly increased in the synbiotic-treated samples (45).

We additionally observed statistically significant, shared decreases in the relative abundance of specific genera or species in both the alcohol and antibiotic treatments (Tables S1 and S2). These included members of the genus *Enterocloster*, which contains many human pathogens, and Bilophila wadsworthia, which is associated with gas and bloating (46). Finally, alcohol and antibiotics additionally yielded compositional effects specific to each stressor following treatment with the synbiotic. For example, the relative abundances of multiple *Faecalibacterium* species, known butyrate producers present in the healthy gut microbiome, were significantly increased in the samples treated with both alcohol and the synbiotic.

Notably, we observed an enrichment of bacterial species involved in the transformation of dietary polyphenols into beneficial urolithin compounds (47) for the antibiotic-plus-synbiotic treatment relative to antibiotic treatment alone. There was an increased abundance of Gordonibacter pamelaeae, which is known to produce urolithin A (48). Additionally, the abundance of Ellagibacter isourolithinifaciens, an organism shown to metabolize intermediate urolithin compounds into the more bioavailable beneficial metabolite isourolithin A (49), was increased in the synbiotic supplementation group after antibiotic treatment (Fig. 2D). We note that the only source of exogenous polyphenols added to the reactors derives from the polyphenol-rich Indian pomegranate extract that comprised the prebiotic component of the synbiotic.

**FIG 3:**
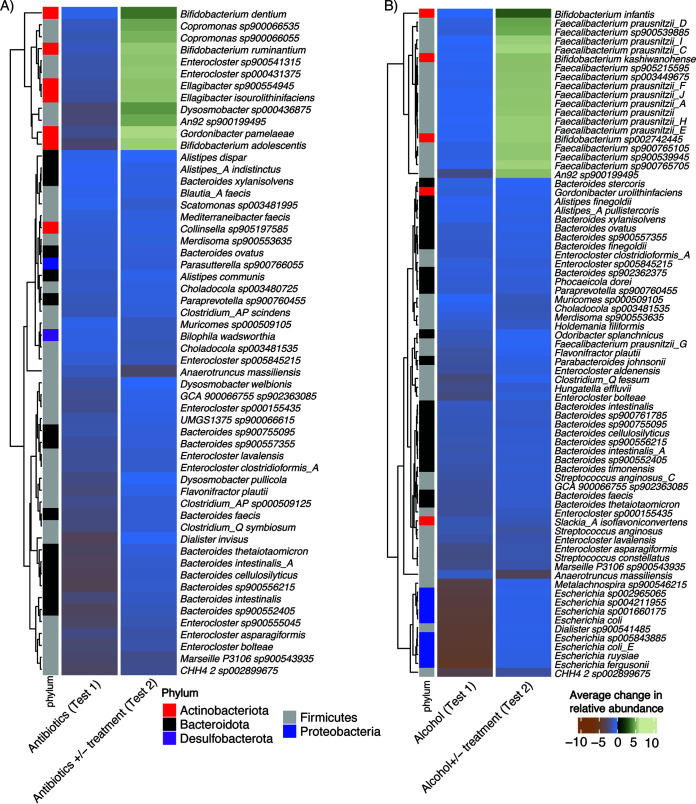
Impact of synbiotic treatment on the relative abundance of a subset of beneficial microbes reduced by stressors. (A) Antibiotics. (B) Alcohol. The left columns in each panel indicate groups with decreased relative abundances in experiment 1 (when reactors were treated with just stressors). The right columns indicate the average shift in abundance for the same microbes that achieved statistical significance (adjusted *P* value of <0.05) in the second experiment, when reactors were treated with stressors with or without a microbial synbiotic. Green represents microbes that were increased in relative abundance after treatment with the synbiotic, while blue/orange indicates microbes that were decreased in relative abundance after treatment with the synbiotic.

Finally, we aimed to determine if synbiotic treatment correlated with the proliferation of any potential pathobionts that were initially repressed by either alcohol or antibiotic usage (Fig. 3A and B). The intent here was to search for evidence of potentially adverse compositional effects that could be induced by treatment. We did not observe this effect. Many species that were initially decreased following treatment with a dysbiotic stressor were further decreased in relative abundance following treatment with a synbiotic. Following antibiotic treatment, for example, organisms that were decreased in relative abundance included Bilophila wadsworthia and many *Bacteroides* strains. For alcohol, the abundances of many Escherichia coli strains were decreased in both experiments.

Certain bacteria that were reduced following initial exposure to a stressor increased in abundance following synbiotic treatment, indicating at least some form of recovery, but not enough for a complete return to baseline abundances (Fig. 3). Some of these were organisms in the synbiotic itself (e.g., Bifidobacterium infantis). Others, however, like Gordonibacter pamelaeae, various *Faecalibacterium* species, and Bifidobacterium dentium, were not present in the synbiotic but still increased in relative abundance.

## DISCUSSION

Our results indicate that a synbiotic may be able to ameliorate microbiome disruptions caused by exposure to antibiotic and alcohol stressors. We identified increased SCFA levels following synbiotic treatment, specifically butyrate, acetate, and lactate, during recovery from stress. We additionally observed higher levels of SCFA-producing and urolithin-producing bacteria. Detrimental or pathogenic microbes (e.g., *B. wadsworthia*) that were depleted following antibiotic or alcohol supplementation were not increased following synbiotic treatment, whereas some beneficial organisms (e.g., *F. prausnitzii* and G. pamelaeae) that were lost following treatment with a stressor were increased in relative abundance after synbiotic treatment.

In this study, synbiotic treatment yielded recoveries in markers of healthy microbiome function, even while the community shifted to an alternate state after treatment. Moreover, in addition to promoting the relative abundance of beneficial, probiotic organisms, treatment with the synbiotic did not result in an increase in known pathobiont taxa. A compositional metric evaluating the “return to baseline” as a surrogate for recovery would not capture these beneficial functional alterations. One key example involves increases in the abundances of organisms known to produce urolithin A, a compound with reported beneficial effects on human aging and mitochondrial function (48, 50). Notably, urolithin A is produced by the fermentation of polyphenols, such as the pomegranate extract found in the synbiotic supplement used in this experiment. The increased abundances of urolithin-producing microbes such as Gordonibacter urolithinfaciens, Gordonibacter pamelaeae, and *Ellagibacter isourolithinifaciens* in the antibiotic treatment following the administration of the synbiotic may therefore reflect native microbial utilization of the supplied prebiotic substrate. It will be of interest to assess whether increased urolithin production is also observed in a clinical setting following the administration of a synbiotic containing a pomegranate-derived prebiotic compound. Conversely, pathobiont-containing genera (e.g., some Escherichia and *Enterocloster* strains) that were initially present at high abundances in the control samples were not increased with synbiotic usage. These observations of beneficial functional changes after synbiotic treatment *in vitro* suggest that metrics for microbiome recovery after dysbiotic stress should include functional changes rather than diversity and taxonomic composition alone.

Our primary heuristic for functional recovery was SCFA production in the presence and absence of synbiotic treatment. Synbiotic treatment stimulated butyrate production that exceeded that of the control fermentations. Butyrate is an important energy source for colonocytes, has anti-inflammatory functions, and is protective against colon cancer and obesity-related disorders (51–54). In contrast to butyrate, propionate levels decreased upon synbiotic treatment. Lower propionate levels may have resulted from decreased abundances of *Bacteroides* spp. and members of the family *Veillonellaceae*, which produce propionate through the direct degradation of complex carbohydrates and the conversion of lactate, respectively (55).

Importantly, we also observed increased acetate levels following synbiotic treatment. Acetate is thought to modulate many aspects of host physiology, ranging from enhancing endurance to protecting against pathobionts (56, 57). Recent work has shown that alcohol consumption stimulates acetate production by the gut microbiome *in vivo* without direct microbial metabolism of ethanol itself (58). While we did not test this hypothesis explicitly, our results are consistent with the observation in a previous study by Martino et al. (58) that alcohol treatment alone did not increase acetate production in an *ex vivo* system. Our observation that acetate was increased in the synbiotic-treated community after alcohol treatment supports the interpretation that either the synbiotic stimulated the growth of SCFA-producing species or species within the synbiotic produced SCFAs.

Our study has certain limitations and leaves a number of open questions. First, it uses an *in vitro* system as a model to approximate the human gut. This artificial system can introduce confounders into the model that make biological interpretations difficult. However, we contend that *in vitro* models using complex fecally derived communities can provide useful information regarding the short-term transit and activity of a synbiotic in the gastrointestinal tract, noting, however, that the use of a fecal community from a single individual does not capture interindividual variations in microbiome composition that may play a role in stress recovery. Second, our incubation times reflect roughly a 2-day exposure time and do not allow conclusions to be drawn on the long-term impact of synbiotics on recovery after stressors. Finally, transfer between reactors induces a bottleneck event that may modify community succession in a manner that would not occur in a true gut ecosystem. Nevertheless, the results shown here indicate the beneficial effects of a specific synbiotic product on the functional recovery of the gut microbiome *in vitro* following stress.

Overall, these experiments demonstrate the acute impact of alcohol and broad-spectrum antibiotics on the fecal microbiome *in vitro* and the potential for a microbial consortium to mitigate environmentally induced stress on the structure and function of the human gut microbiome *in vivo*. We contend that our results demonstrating the recovery of key aspects of healthy gut microbial function, specifically SCFA production, are relevant to predicting the potential effects of a synbiotic in humans. A well-designed clinical trial with deeper sequencing, additional measurements of microbial function, and a larger sample size will help to test the broader applicability of these findings and the role of synbiotics in the recovery of the human gut microbiome following exposure to stressors.

## MATERIALS AND METHODS

### Antibiotics, alcohol, and microbial consortium.

Metronidazole and ciprofloxacin were obtained from Sigma-Aldrich (Bornem, Belgium), while vodka was used as the alcohol (40% [vol/vol] EtOH) source. SH-DS01 capsules containing a 24-strain probiotic mixture encased in an Indian pomegranate (Punica granatum) outer envelope were obtained from Seed Health (Los Angeles, CA, USA). SH-DS01 contains the following strains: Bifidobacterium breve HRVD521-US, *Lacticaseibacillus rhamnosus* HRVD113-US, Bifidobacterium lactis HRVD524-US, *Lacticaseibacillus casei* HRVD300-US, Bifidobacterium longum SD-BB536-JP, *Lactiplantibacillus plantarum* SD-LPLDL-UK, Bifidobacterium animalis subsp. *lactis* SD-MB2409-IT, Bifidobacterium longum SD-CECT7347-SP, *Lacticaseibacillus casei* SD-CECT9104-SP, Bifidobacterium lactis SD-CECT8145-SP, *Lactiplantibacillus plantarum* SD-LP1-IT, Bifidobacterium breve SD-BR3-IT, Lacticaseibacillus rhamnosus SD-LR6-IT, Limosilactobacillus reuteri SD-LRE2-IT, Bifidobacterium infantis SD-M63-JP, Bifidobacterium longum HRVD90b-US, Bifidobacterium lactis SD-BS5-IT, Bifidobacterium lactis SD150-BE, *Limosilactobacillus reuteri* SD-RD830-FR, *Lacticaseibacillus rhamnosus* SD-GG-BE, Bifidobacterium adolescentis SD-BA5-IT, Lactobacillus crispatus SD-LCR01-IT, Limosilactobacillus fermentum SD-LF8-IT, and Ligilactobacillus salivarius SD-LS1-IT.

### Establishment of dysbiotic conditions.

A batch fermentation approach was used for this study. An initial test with fecal material from two healthy individuals was conducted to optimize the dysbiotic conditions. Healthy donors, one male and one female, between 29 and 35 years of age, with a body mass index (BMI) of between 19 and 25, without antibiotic consumption in the previous 6 months or alcohol consumption in the previous 3 days, and with a self-selected standard Western diet were used for this research. Ethical approval by the University Hospital Ghent (reference no. B670201836585) was obtained for the collection of fecal samples, and written informed consent was obtained prior to sample collection. In this prescreening step, colonic bioreactors were inoculated with nutritional medium representative of the human colon (1 g/L of starch [Carl Roth, Karlsruhe, Germany], glucose [Merck KGaA, Darmstadt, Germany], pectin, Arabic gum [Keyser & Mackay, Amsterdam, The Netherlands], and fructooligosaccharides [FOSs] [Sigma-Aldrich, Bornem, Belgium]; 1.8 g/L yeast extract and peptone [Oxoid, Aalst, Belgium]; 4.7 g/L K_2_HPO_4_; 14.7 g/L KH_2_PO_4_; 1.8 g/L NaHCO_3_ [Chem-lab NV, Zedelgem, Belgium]; 0.45 g/L l-cysteine; and 1.8 mL/L Tween 80 [Sigma-Aldrich, Bornem, Belgium] [pH 6.5]) and five mucin-coated carriers (meant to simulate a healthy gut mucosal lining), prepared according to methods described previously by Van den Abbeele et al. (44).

K1 carriers (AnoxKaldnes AB, Lund, Sweden) were submerged in a mucin solution composed of 50 g/L gastric porcine mucin type II (Sigma-Aldrich) and 10 g/L bacteriological agar (Oxoid) and combined in polyethylene netting (Zakkencentrale, Rotterdam, The Netherlands). Subsequently, anaerobic conditions were obtained by flushing bioreactors with N_2_ for 10 min, and fecal samples were inoculated with a 10% (vol/vol) fecal slurry obtained by mixing the fecal sample with 7.5% (wt/vol) anaerobic phosphate-buffered saline (PBS), as previously described by Calatayud et al. (59). For both donors, a control condition, 2 doses of vodka (10% and 30% [vol/vol], corresponding to 4% and 12% EtOH, respectively), and 2 doses of an antibiotic mixture (50 μg/mL metronidazole plus 30 μg/mL ciprofloxacin and 250 μg/mL metronidazole plus 164 μg/mL ciprofloxacin) were tested. Different treatments were added to the reactors and incubated at 37°C for 48 h under anaerobic conditions. After this time, luminal samples were obtained to assess the overall microbial fermentation (pH) and microbial metabolic activity in terms of the production of saccharolytic end products (SCFAs) (see Table S1 in the supplemental material). The conditions selected had the highest microbial metabolic activity following treatment. These were low-dose antibiotics and 30% (vol/vol) vodka.

### Experimental design.

For the final experiment, two subsequent batch incubations were performed, focusing on (i) dysbiosis induction and (ii) recovery assessment. The total volume per reactor was 70 mL. During the first 48-h incubation (“dysbiosis induction”), three test arms were run in parallel: the untreated control, antibiotic treatment (50 μg/mL metronidazole and 30 μg/mL ciprofloxacin), and ethanol treatment (30% [vol/vol] vodka). The nutritional medium and inoculation density were identical to the ones specified above. Subsequently, at the start of the second incubation (“recovery assessment”), 10% (vol/vol) of the preceding 48-h control-, antibiotic-, or ethanol-treated microbiome was added to fresh nutritional medium. Furthermore, to increase the biorelevance of the recovery assessment, precolonized mucin-coated carriers from the untreated control were administered (Fig. 1A). This simulates recolonization by gut microbes from environments (such as the biofilm covering the mucosal surface) where microbes are better protected from external stressors such as antibiotics and ethanol. The test arms during the recovery assessment comprised the following: (i) control plus mucin beads, (ii) antibiotic treatment plus mucin beads, (iii) alcohol treatment plus mucin beads, (iv) antibiotic treatment plus mucin beads and 5.3 × 10^10^ arbitrary fluorescence units (AFU) of the 24-strain probiotic, and (v) alcohol treatment plus mucin beads and 5.3 × 10^10^ AFU of the 24-strain probiotic. Reactors were incubated anaerobically at 37°C for a further 48 h. All of the above-mentioned test arms were run using a fecal sample from one donor (in three technical replicates). Six milliliters was sampled from each reactor for downstream analyses at different time points.

### Microbial metabolic activity analysis.

Samples were collected after further 0-h, 6-h, 24-h, and 48-h colonic fermentations and assessed for metabolic activity by measuring acetate, propionate, and butyrate as previously described by De Weirdt et al. (60). Previous work demonstrated that fecal suspensions are active for up to 48 h in the medium used here (61). Briefly, after the addition of 2-methyl hexanoic acid as an internal standard, SCFAs were extracted from the samples with diethyl ether. Extracts were analyzed using a GC-2014 gas chromatograph (Shimadzu, ‘s-Hertogenbosch, The Netherlands) equipped with a capillary fatty-acid-free EC-1000 Econo-Cap column (dimensions of 25 mm by 0.53 mm, film thickness of 1.2 μm; Alltech, Laarne, Belgium), a flame ionization detector, and a split injector. The production of unbranched and branched SCFAs was calculated by summing the molar concentrations of acetate, propionate, butyrate, valerate, and caproate and summing the molar concentrations of isobutyrate, isovalerate, and isocaproate, respectively. Total SCFA production was defined as the sum of unbranched and branched SCFAs. Lactate was quantified using a commercially available enzymatic assay kit (R-Biopharm, Darmstadt, Germany).

### Identification of microbial community relative abundances.

DNA was isolated as previously described (62). Bacterial cell pellets, collected from 1-mL luminal colonic suspensions, were mechanically and chemically lysed using glass beads and a cationic detergent (cetyltrimethylammonium bromide), respectively. DNA was extracted according to a phenol-chloroform-isoamyl alcohol protocol and precipitated with polyethylene glycol. Finally, the DNA was washed with 70% alcohol and diluted in DNase/RNase/protease-free water. Samples were shipped to CosmosID Inc. (Rockville, MD, USA) for shallow shotgun sequencing analysis. For this purpose, DNA libraries were prepared using the Illumina Nextera XT library preparation kit, with a modified protocol. The library quantity was assessed with a Qubit instrument (Thermo Fisher). Libraries were then sequenced on an Illumina HiSeq 2× 150-bp platform at 3 million total reads. Raw reads were quality controlled by CosmosID to remove trim adapters and human contamination.

Relative abundances were computed from the sequencing output. The quality-controlled reads were aligned back to the Genome Taxonomy Database (GTDB) (release 207) (63) with the BURST package (64). The GTDB was formatted with the BURST plug-in xtree with a kmer length of 29 and a compression level set to 2. Alignments were filtered based on coverage, keeping only genomes with either 0.02% unique coverage (across all genomes in the database) or 50% total. Relative abundance was computed via center-log-ratio transformation. As a secondary approach, we computed relative abundances by summing the total numbers of reads aligned to all genomes in a given sample and dividing the reads aligned to a given genome by this sum (Table S2).

### Statistics.

The following comparisons were made between the different arms of the experiment. For test 1 (Fig. 1A, top row), comparisons were made for (i) the control versus the antibiotic-treated reactor (Fig. 1C) and (ii) the control versus the EtOH-treated reactor (Fig. 1C). For test 2 (Fig. 1A, bottom row), comparisons were made for (i) the antibiotic-treated reactor with synbiotic treatment versus the antibiotic-treated reactor without synbiotic treatment (Fig. 2D), (ii) the EtOH-treated reactor with synbiotic treatment versus the EtOH-treated reactor without synbiotic treatment (Fig. 2D), and (iii) both antibiotics and ethanol reactors without synbiotic treatment versus both antibiotics and ethanol reactors with synbiotic treatment (Fig. 3).

All comparisons between groups were carried out using generalized linear regression on center-log-ratio-transformed raw counts (figures in the text) or relative abundances (see the supplemental material).

### Data availability.

The data that support the findings of this study are openly available in the National Center for Biotechnology Information (NCBI) repository under accession no. PRJNA689370.
